# Supplementary material for: Autonomous mesoscale positioning emerging from myelin filament self-organization and Marangoni flows
Source: Nat Commun. 2020 Sep 23;11:4800. doi: 10.1038/s41467-020-18555-w (PMC7511956; doi:10.1038/s41467-020-18555-w)
Supplement: Supplementary file 1 — Supplementary Information [file 41467_2020_18555_MOESM1_ESM.pdf]

Autonomous mesoscale positioning emerging from myelin filament self-organization and Marangoni flows

Van der Weijden et al.

Supplementary Figures

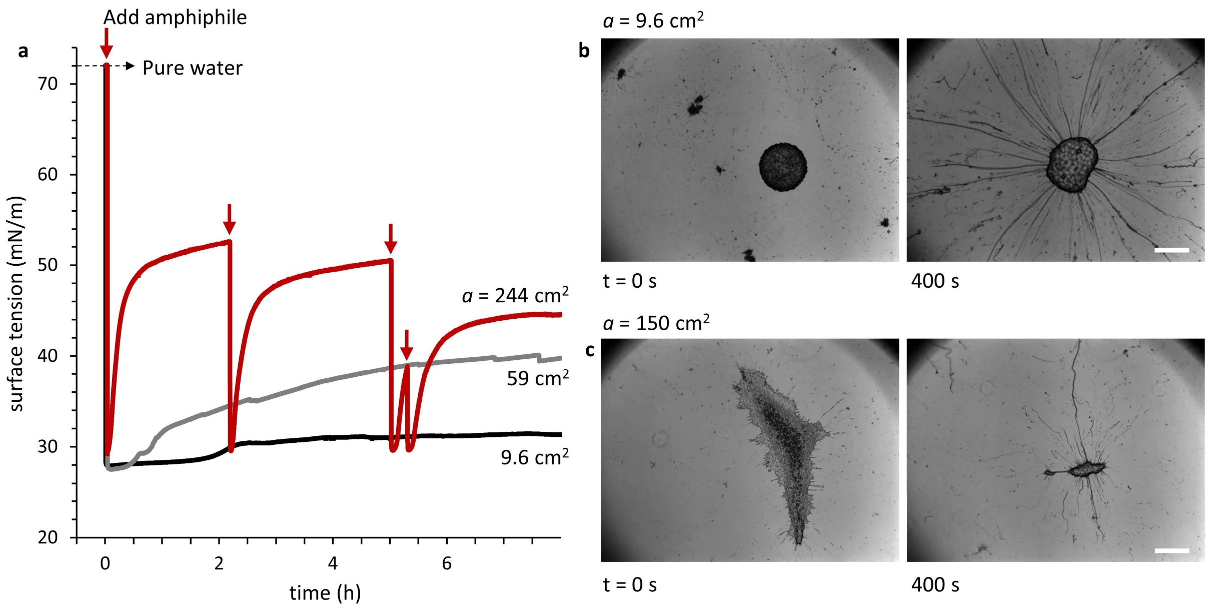

Supplementary Figure 1. The effect of the air-water interface area on the amphiphile depletion rate. a, Surface tension *vs*. time, measured upon depositing an amphiphile source solution droplet (1.0 μL) at t = 2 min at the interface of pure water. The decrease in surface tension demonstrates the release of individual amphiphiles from the source droplet; the subsequent recovery indicates a depletion of these amphiphiles from the air-water interface to the water bulk phase. The red arrows indicate the time points of deposition of new amphiphile solution droplets (1.0 μL). Upon decreasing the air-water interface area from a = 244 cm^2 to a = 59 cm^2 and a = 9.6 cm^2, the surface tension recovery is retarded, since a larger amount of amphiphile molecules is available per square centimetre to replace the depleted amphiphiles. **b-c,** Optical microscopy recording of amphiphile source droplets (1.0 μL) deposited at t = 0 s on pure water with an air-water interface of a = 9.6 cm^2 (b), and a = 150 cm^2 (c), revealing that the enhanced overall depletion rate of the amphiphile towards the aqueous phase from a larger air-water interface gives a faster extrusion of filaments that finishes the source droplet faster as well. The scale bars represent 1 mm.

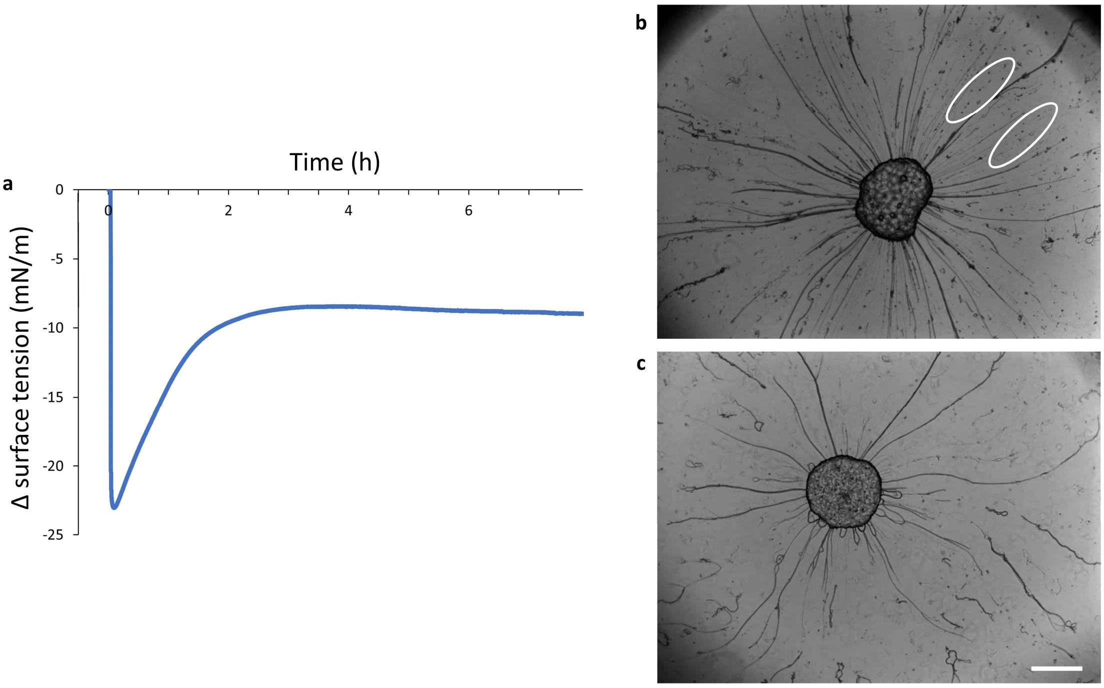

Supplementary Figure 2. Influence of sodium alginate on amphiphile depletion kinetics and filament stability. a, Change in surface tension *vs*. time, measured upon depositing an amphiphile solution droplet (1.0 µL) at t = 2 min at the interface of an aqueous sodium alginate solution (6.25 mg/mL) with an area of a = 244 cm^2. **b-c,** Optical microscopy recording of an amphiphile source droplet floating at a pure water solution (b) and an aqueous sodium alginate solution (6.25 mg/mL, c), respectively (a = 9.6 cm^2). We noted that the presence of sodium alginate enhances the stability of the filaments; when no sodium alginate is present, the filaments fall apart more rapidly, as can be seen from the larger amount of small particles (exemplified by white ovals in b). The scale bar represents 1 mm.

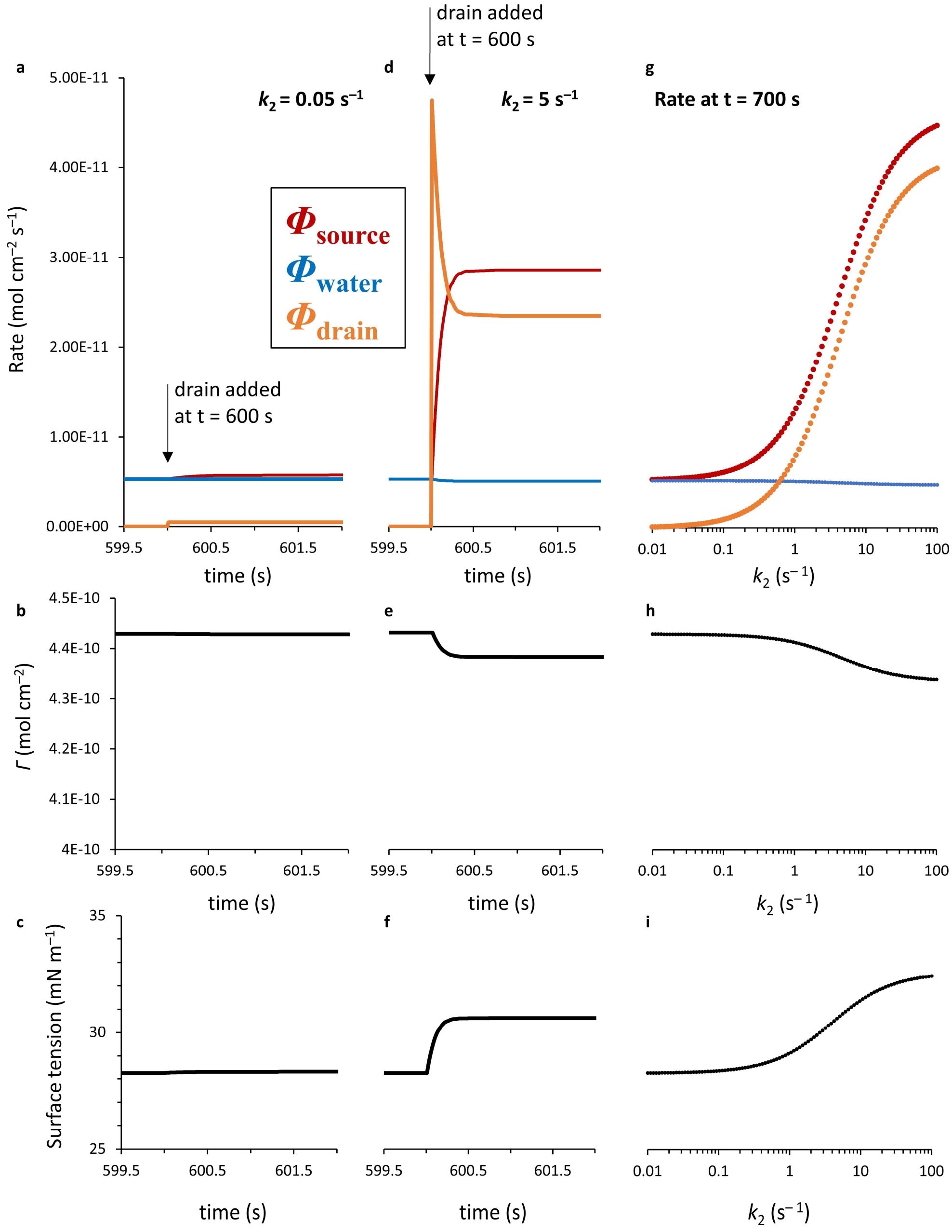

Supplementary Figure 3. Simulations with the kinetic model. We analyze the effect of drain deposition on the rates of: amphiphile release from the source droplet (Φ_{source}, in mol cm^{-2} s^{-1}); depletion towards the drain droplet (Φ_{drain}) and depletion towards the underlying aqueous phase (Φ_{water}). **a,** Simulation of Φ_{source}, Φ_{drain}, and Φ_{water} *vs*. time upon deposition of a "weak" drain at t = 600 s (*i.e.* switch from $k_2 = 0$ to $k_2 = 0.05$ s^{-1}). Under these conditions, $\Phi_{source} \approx \Phi_{water} >> \Phi_{drain}$ and both the amphiphile density at the air-water interface Γ (**b**) and the surface tension (**c**) remain constant after deposition of the drain. **d,** Simulation of Φ_{source}, Φ_{drain}, and Φ_{water} *vs*. time upon deposition of a "strong" drain at t = 600 s (*i.e.* switch from $k_2 = 0$ to $k_2 = 5$ s^{-1}). Under these conditions, $\Phi_{source} \approx \Phi_{drain} > \Phi_{water}$ and the enhanced depletion of amphiphile from the air-water interface decreases Γ (**e**) and increases the surface tension (**f**). **g,** The time-dependent Φ_{source}, Φ_{drain}, and Φ_{water} were simulated with the kinetic model, using different values of k_2. The plot shows, as a function of k_2, the values of Φ_{source}, Φ_{drain}, and Φ_{water} 100 seconds after the deposition of a drain (at t = 600 s). **h,** Γ, 100 seconds after deposition of the drain *vs*. k_2. **i,** Surface tension, 100 seconds after deposition of the drain *vs*. k_2. The conditions for the simulations, as well as the values of parameters not shown here are discussed in the Methods section.

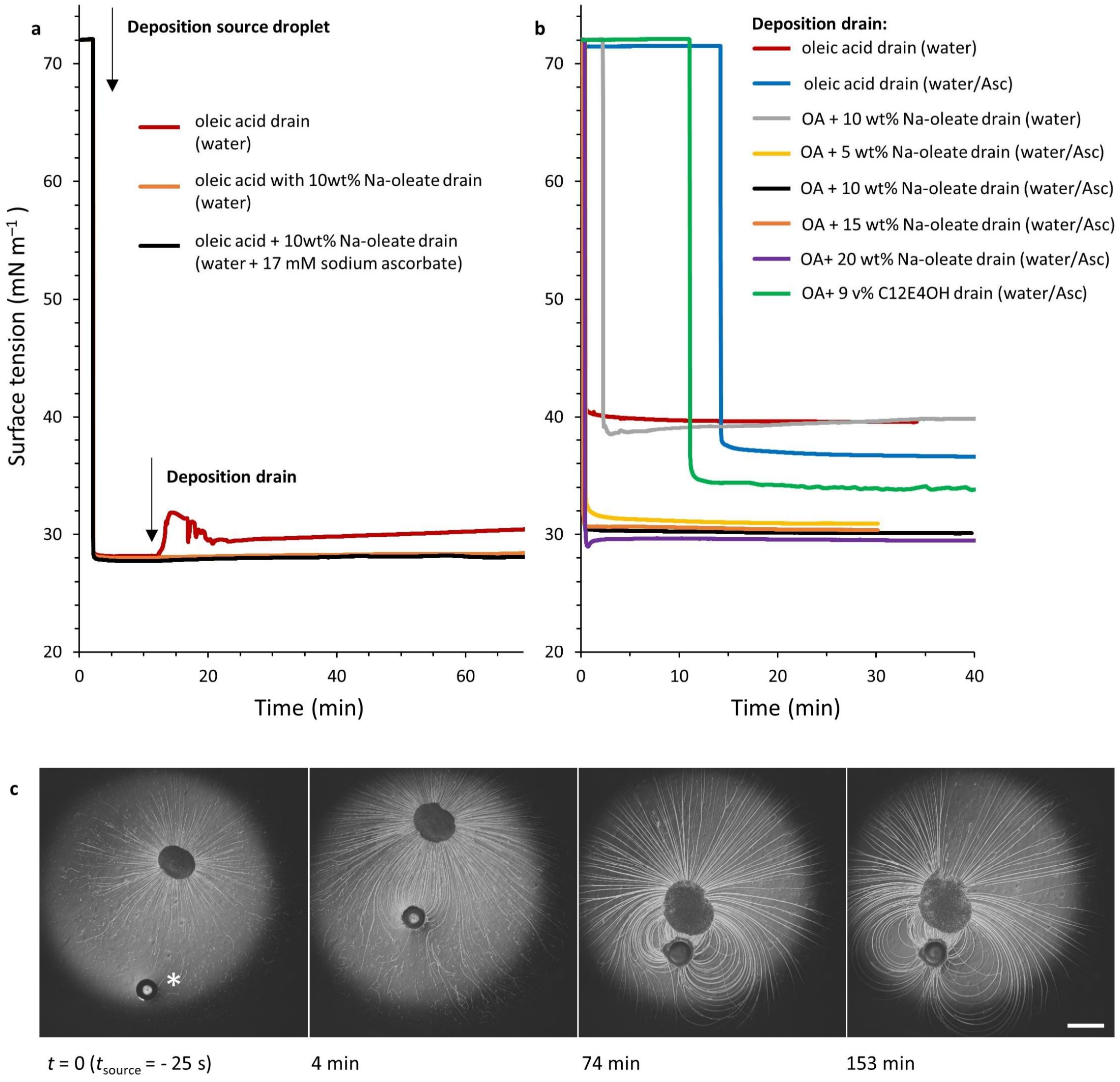

Supplementary Figure 4. Effect of surfactant included in the drain droplet on depletion kinetics. a, Experimentally observed surface tension *vs*. time. At $t = 2$ min, an amphiphile solution droplet (0.5 µL) was deposited at the air-water interface, at $t = 12$ min, the respective drain droplet (0.5 µL) was deposited: pure oleic acid and oleic acid with 10 wt% sodium oleate, respectively. Upon deposition of the oleic acid drain, the surface tension increased, indicating the depletion at the drain is faster than the depletion to the underlying aqueous phase ($\Phi_{\mathrm{source}} \approx \Phi_{\mathrm{drain}} > \Phi_{\mathrm{water}}$). With sodium oleate included in the drain, the amphiphile uptake slowed down and the surface tension remained constant when the drain was deposited, indicating that the drain only provided a minor addition to the original depletion of $C_{12}E_4OH$ from the air-water interface, and $\Phi_{\mathrm{source}} \approx \Phi_{\mathrm{water}} >> \Phi_{\mathrm{drain}}$. Similar behavior was observed when the experiment with a 10 wt% sodium oleate in oleic acid drain was conducted on an aqueous solution that contained 17 mM sodium ascorbate. **b,** Experimentally observed surface tension *vs*. time, upon deposition of drain droplets (1.0 µL) with different compositions at the air-water interface of pure water and an aqueous 17 mM sodium ascorbate (water/Asc) solution, respectively. Importantly, for all drain droplet compositions (oleic acid, sodium oleate in oleic acid (OA), $C_{12}E_4OH$ in oleic acid), the drop in surface tension upon deposition of the respective drain is insufficient to obtain the surface tension value of 28 mM m^{-1} that was observed when a 10 wt% sodium oleate in oleic acid drain droplet was deposited after the deposition of an amphiphile source droplet (a). **c,** Optical microscopy recording at 1.25x magnification of the self-organization that is established by a pure $C_{12}E_4OH$ source droplet (1.0 µL, deposited at $t_{\mathrm{source}} = -25$ s) with a 9 v/v% $C_{12}E_4OH$ in oleic acid drain droplet (1.0 µL, indicated with asterisk, deposited at $t = 0$ s). The scale bar represents 2 mm.

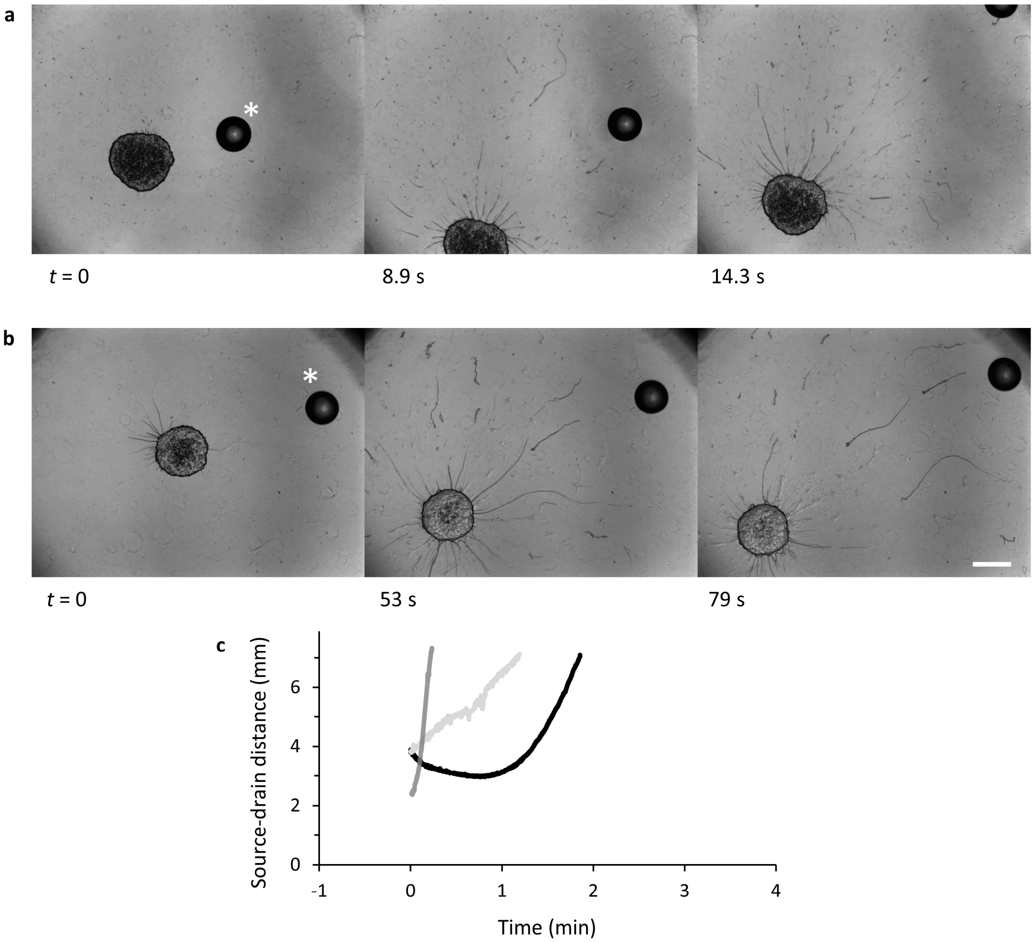

Supplementary Figure 5. Repulsion of drain droplet from the source in regime 2. a-b, Optical microscopy recording of two separate experiments where the Marangoni flow emerging from a free-floating amphiphile source droplet (0.5 μL) pushes a drain droplet (10 wt% sodium oleate in oleic acid, 0.5 μL, indicated with asterisk) away over the air-water interface – according to regime 2 in Figure 2. The drain droplets were deposited at $t = 0$ s. The scale bar represents 1 mm. **c,** Source-drain distance *vs*. time graph, as shown in Figure 2h of the main text: the medium grey curve corresponds to the data shown in a), the light grey curve to the data shown in b) and the black curve to the data shown in Figure 2f.

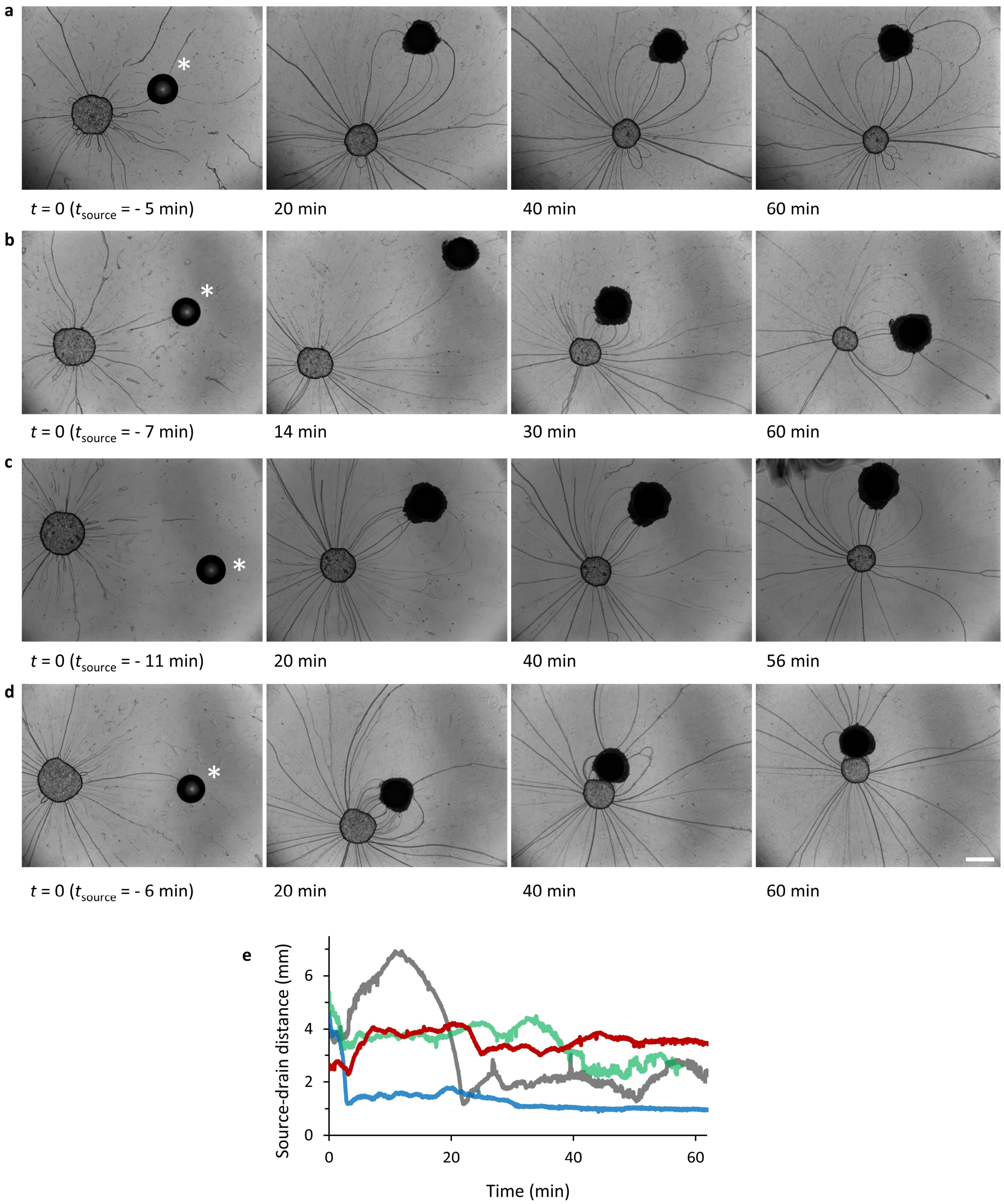

Supplementary Figure 6. Self-positioning of source and drain droplets in regime 3. a-d, Optical microscopy recording of four separate experiments, conducted under similar conditions, where a free-floating amphiphile source droplet (0.5 µL) and drain droplet (10 wt% sodium oleate in oleic acid, 0.5 µL, indicated with asterisk) self-organize at the air-water interface – according to regime 3 in Figure 2. The drain droplets were deposited at $t = 0$ min, the time at which the source droplet was deposited (t_{source}) is shown in brackets. The scale bar represents 1 mm. **e,** Source-drain distance *vs*. time graph, as shown in Figure 2h of the main text. The red curve corresponds to the data shown in a), the grey curve to the data shown in b), the green curve to the data shown in c) and the blue curve to the data shown in d).

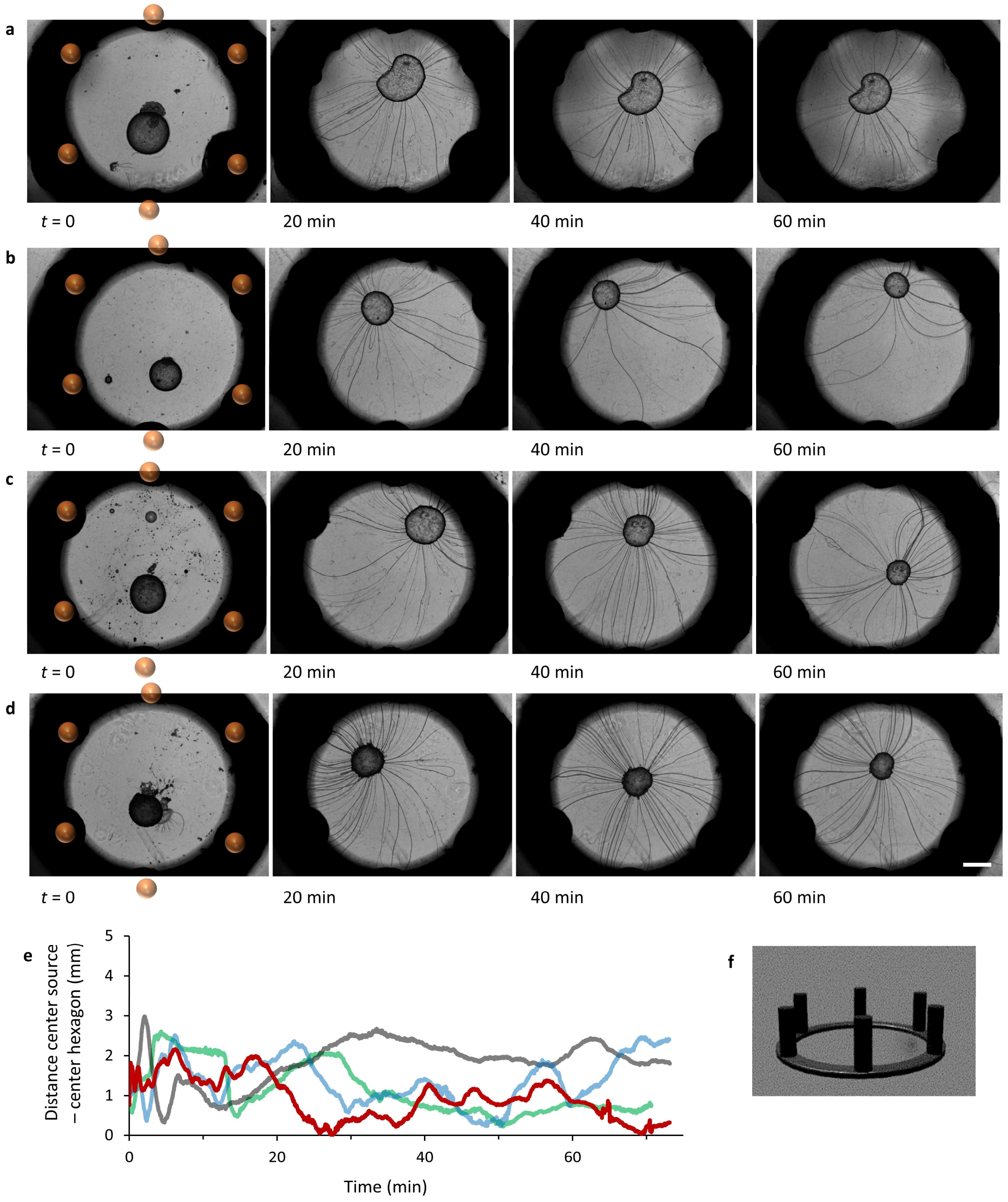

Supplementary Figure 7. Autonomous positioning of source droplet in a hexagonal ring of drain droplets. a-d, Optical microscopy recording of four separate experiments, conducted under similar conditions, where a free-floating amphiphile source droplet (1.0 µL, deposited at t = 0 s) maintains its position in between six drain droplets (10 wt% sodium oleate in oleic acid, 1.0 µL, indicated with orange spheres in the left pane) that have been placed at the tips of hexagonally positioned pins on a ring. The scale bar represents 1 mm. **e,** Distance center source – center hexagon *vs*. time graph, as shown in Figure 4f of the main text. The green curve corresponds to the data shown in a), the grey curve to the data shown in b), the blue curve to the data shown in c) and the red curve to the data shown in d). **f,** 3D computer drawing of hexagon with 6 pins; a polylactic acid 3D print of this hexagon was placed in the sodium alginate solution to keep the 6 drain droplets in position.

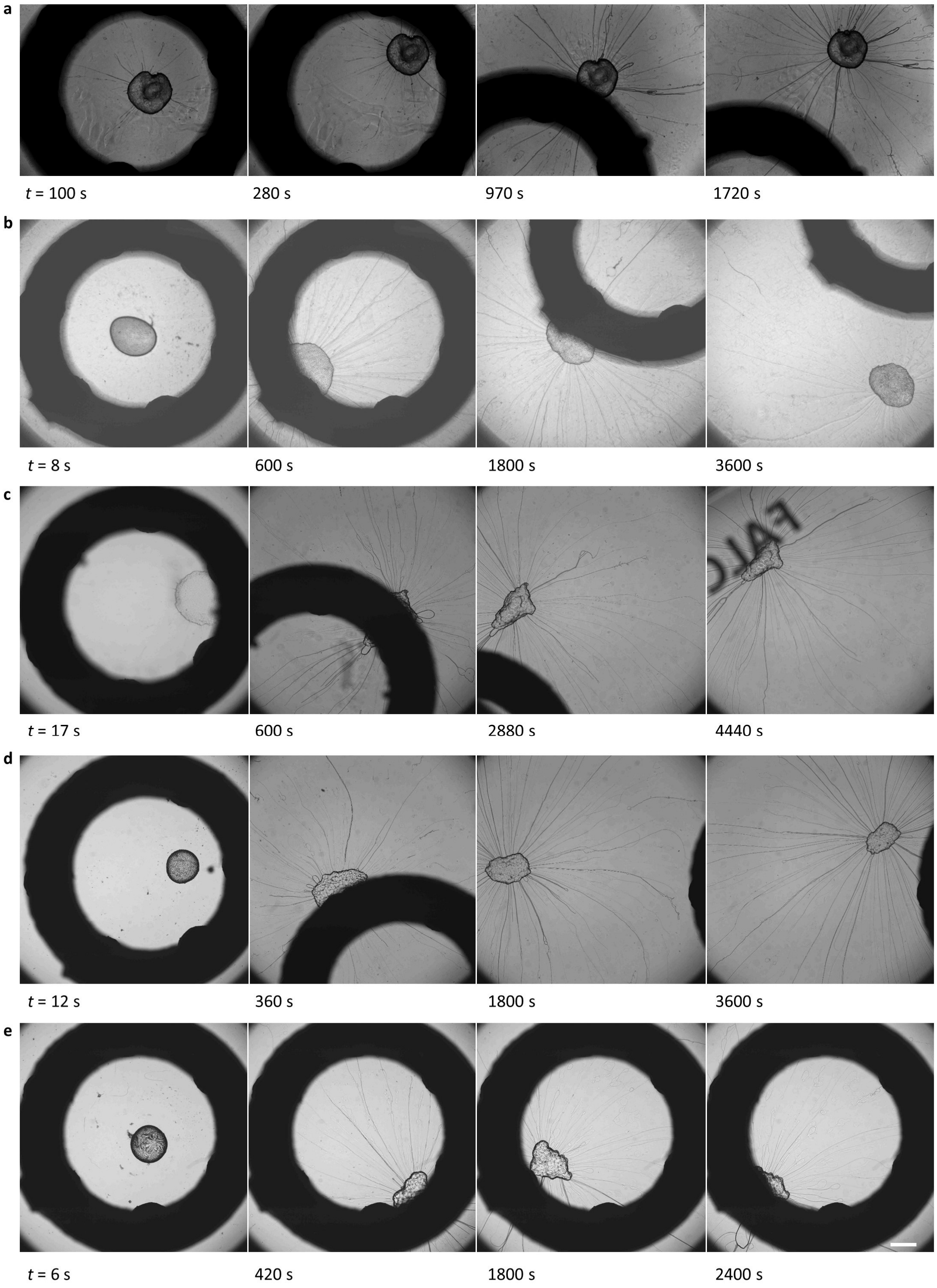

Supplementary Figure 8. Positioning of the source droplet in the absence of drain droplets. a-e, Optical microscopy recording of five separate experiments, conducted under similar conditions, where a free-floating amphiphile source droplet (1.0 μL) was deposited at the air-water interface within the hexagon that was placed in the sodium alginate solution, corresponding to the blue curves in Figure 4f of the main text. Since no drain droplets were deposited at the hexagon pins, the filaments had no adhesion points to keep the source in position, and as a result, the source droplet was able to move around randomly, within and outside of the hexagon. The scale bar represents 1 mm.

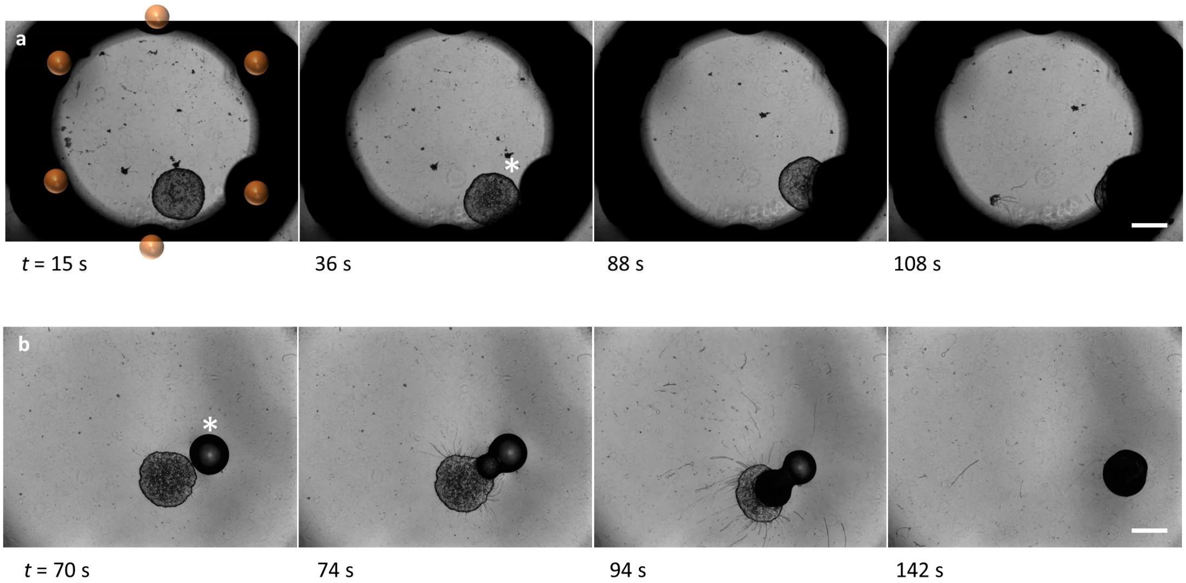

Supplementary Figure 9. Merging of source and drain droplets. When the source and drain droplets get into physical contact before the filament growth has started, merging has been observed. **a,** Optical microscopy recording of a free-floating amphiphile source droplet (1.0 µL) that was deposited at the air-water interface at *t* = 0 s, in between six drain droplets (10 wt% sodium oleate in oleic acid, 1.0 µL, indicated with the orange spheres in the left pane) that have been placed at the tips of hexagonally positioned pins on a ring. After approx. 36 seconds, the source droplet bounced into one of the drain droplets (indicted with asterisk), and merged with this droplet. **b,** Optical microscopy recording of a free-floating amphiphile source droplet (0.5 µL, deposited at *t* = 0 s) and a drain droplet (10 wt% sodium oleate in oleic acid, 0.5 µL, deposited at *t* = 34 s, indicated with asterisk) at the air-water interface. After approx. 70 seconds, the source and drain contacted – before the filament growth started – and the droplets merged. The scale bars represent 1 mm.

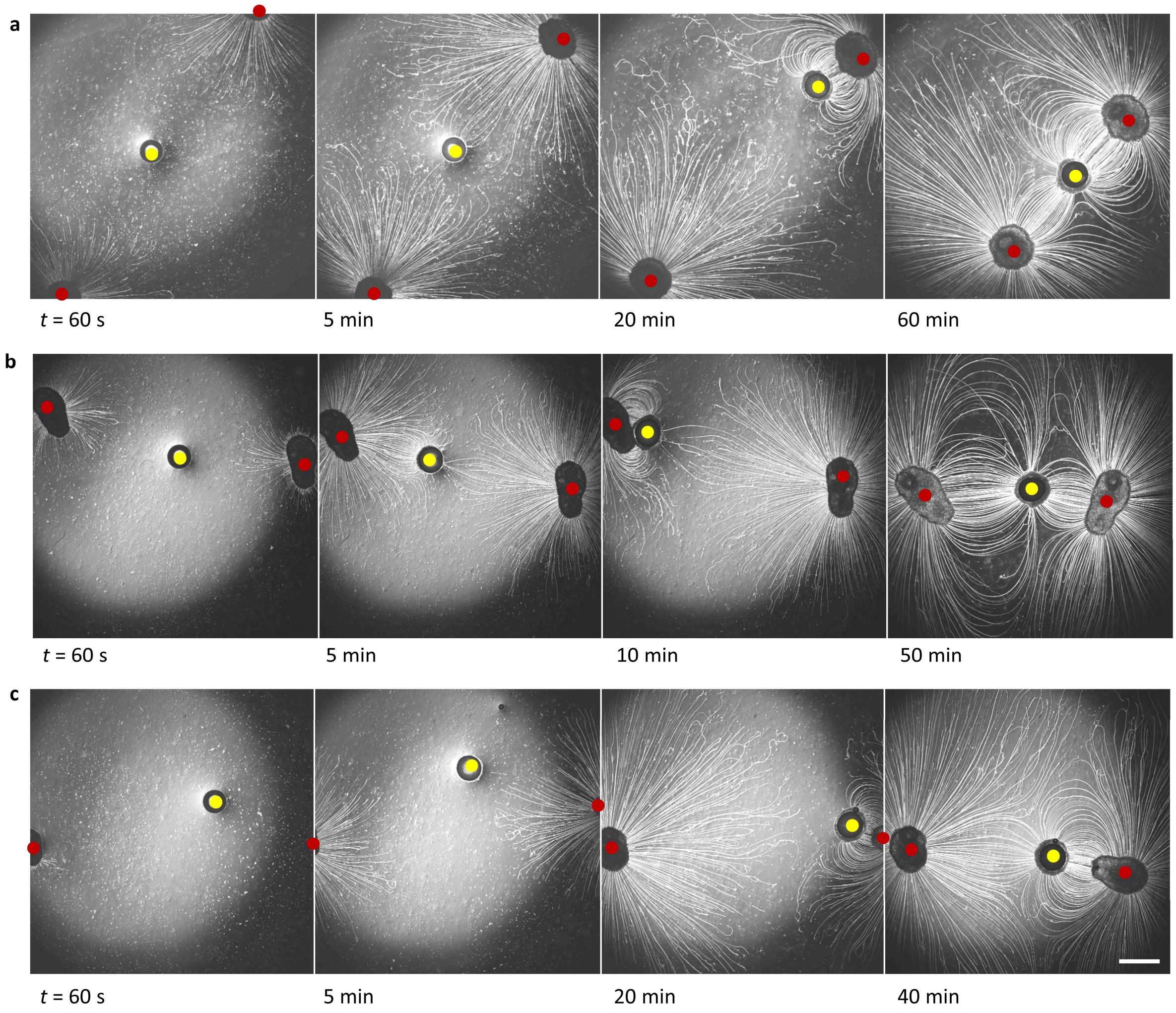

Supplementary Figure 10. Linear self-organization of a 3-droplet system. Optical microscopy recording at 1.25x magnification of 3 separate experiments, conducted under similar conditions, where a drain droplet (10 wt% sodium oleate in oleic acid, 1.0 µL, indicated with the yellow dot) positioned itself in between two free-floating amphiphile source droplets (pure $C_{12}E_4OH$, 1.0 µL, indicated with the red dots) at the air-water interface of the sodium alginate solution. We observed – while allowing the self-organization to take place under similar conditions as in Supplementary Figs. 11-13 and Fig. 6a – the linear self-organization as schematically shown in Fig. 5e for $n = 5$ times (**a**, **b**, **c**, Fig. 6a, Suppl. Fig. 11). The scale bar represents 2 mm.

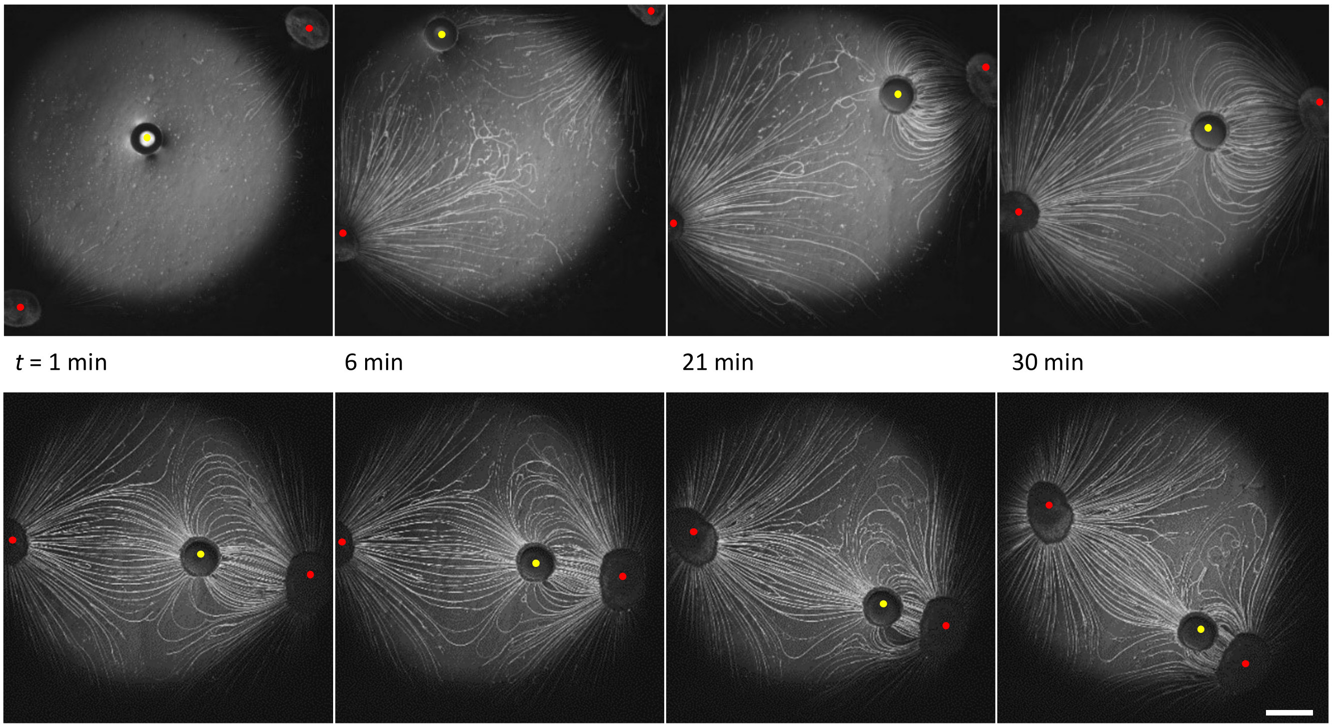

t = 1 min | 6 min | 21 min | 30 min

2 h | 3 h | 4 h | 5.5 h

Supplementary Figure 11. Positioning of source and drain droplets over time period of >5 hours. Optical microscopy recording at 1.25x magnification of a drain droplet (10 wt% sodium oleate in oleic acid, 1.0 µL, indicated with the yellow dot) positioning itself in between two free-floating amphiphile source droplets (pure $C_{12}E_4OH$, 1.0 µL, indicated with the red dots) at the air-water interface of the aqueous sodium alginate solution. The drain droplet was observed to maintain its positioning in between the two source droplets over a time period of more than 5 hours, while attracting filaments that originated from both source droplets. The scale bar represents 2 mm.

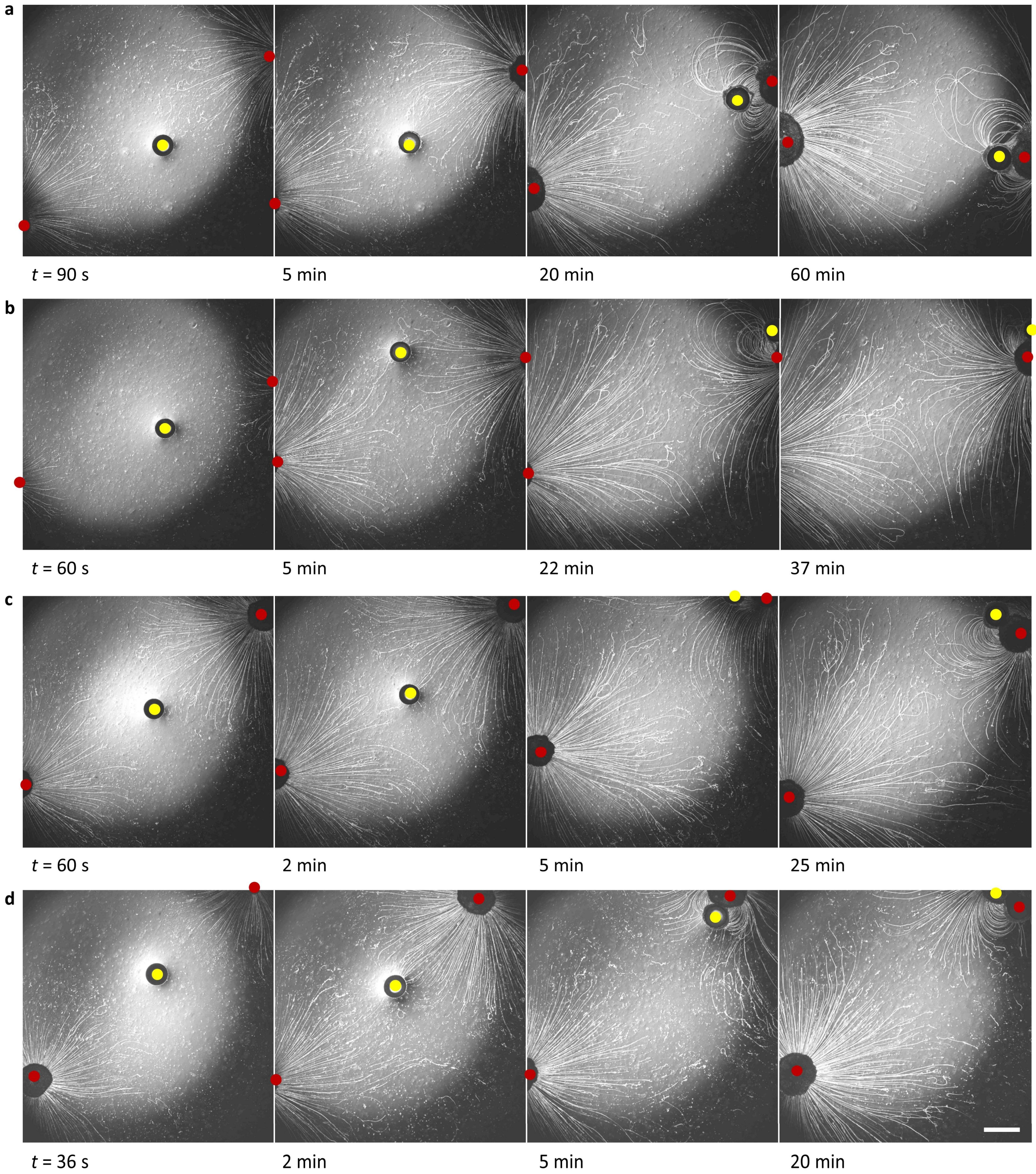

Supplementary Figure 12. Self-organization of a 3-droplet system, with filament obstruction. Optical microscopy recording at 1.25x magnification of 4 separate experiments, conducted under similar conditions, where a drain droplet (10 wt% sodium oleate in oleic acid, 1.0 μL, indicated with the yellow dot) was deposited in between two free-floating amphiphile source droplets (pure $C_{12}E_4OH$, 1.0 μL, indicated with the red dots) at the air-water interface of the sodium alginate solution. We observed – while allowing the self-organization to take place under similar conditions as in Supplementary Figs. 10, 11 and 13 and Fig. 6a – the behavior as schematically shown in Fig. 5c for n = 4 times (**a**, **b**, **c, d**). The scale bar represents 2 mm.

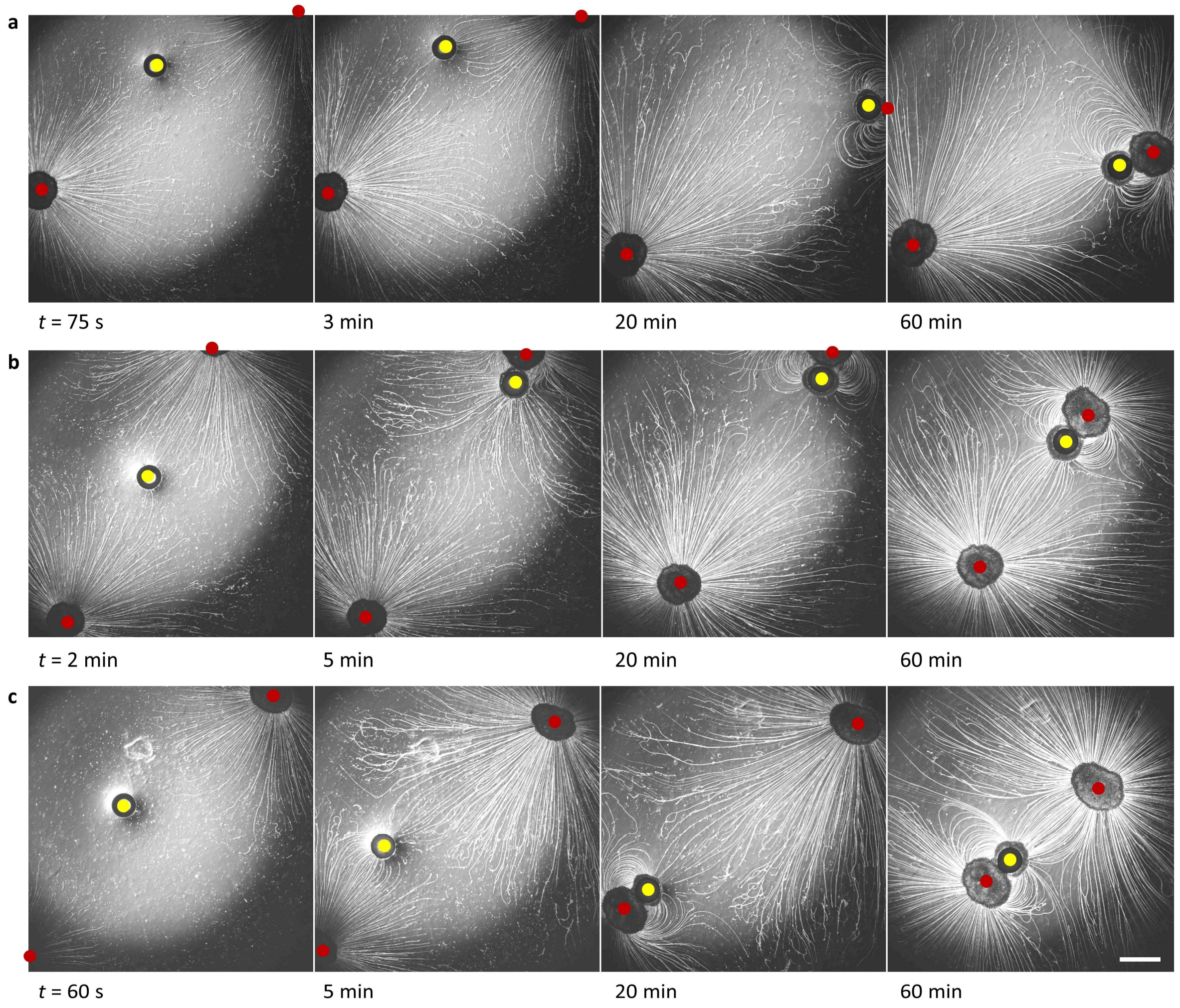

Supplementary Figure 13. Self-organization of a 3-droplet system, where drain sticks to source. Optical microscopy recording at 1.25x magnification of 3 separate experiments, conducted under similar conditions, where a drain droplet (10 wt% sodium oleate in oleic acid, 1.0 μL, indicated with the yellow dot) was deposited in between two free-floating amphiphile source droplets (pure $C_{12}E_4OH$, 1.0 μL, indicated with the red dots) at the air-water interface of the sodium alginate solution. We observed – while allowing the self-organization to take place under similar conditions as in Supplementary Figs. 10-12 and Fig. 6a – the behavior as schematically shown in Fig. 5d for $n = 3$ times (**a**, **b**, **c**). The scale bar represents 2 mm.

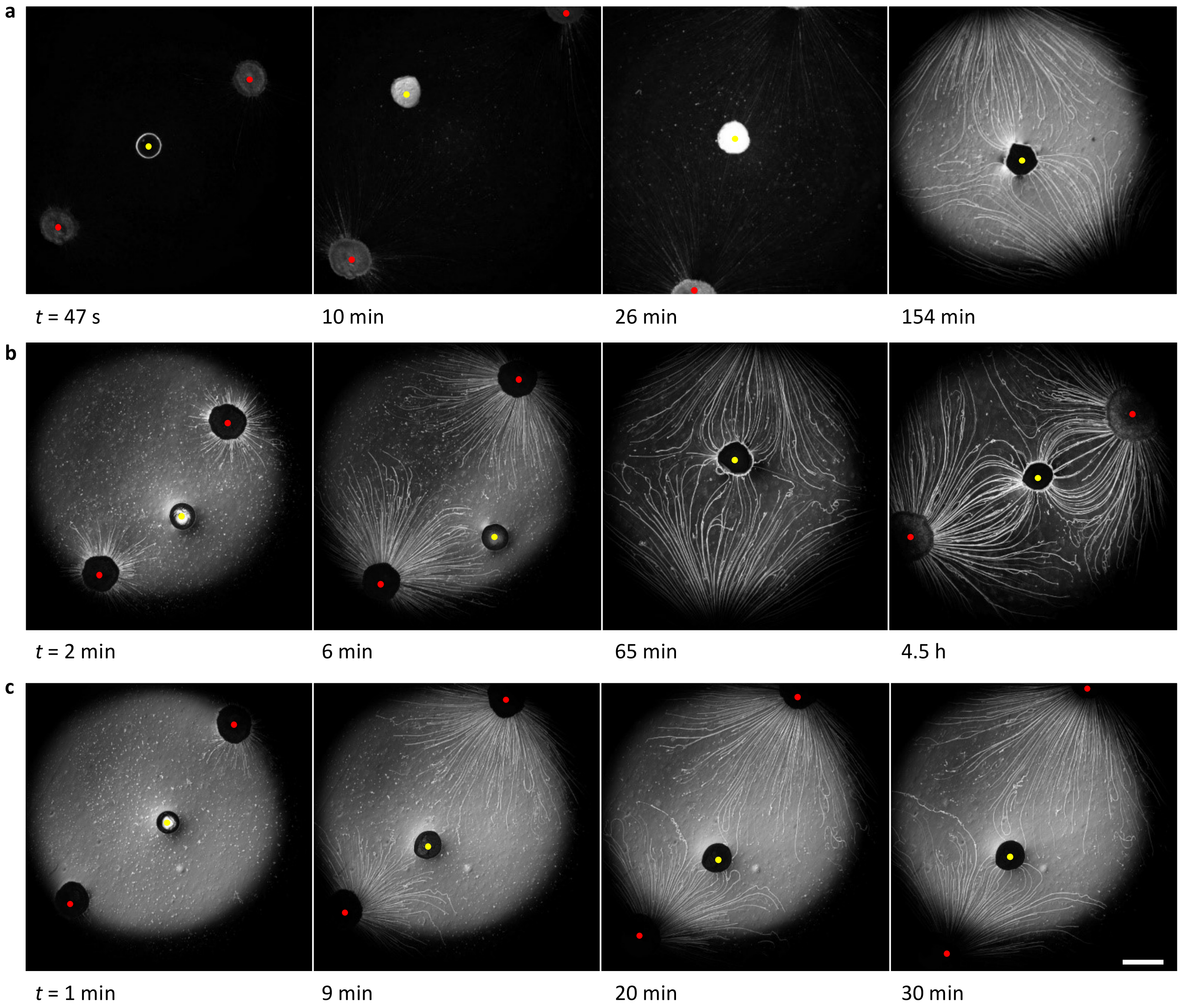

Supplementary Figure 14. Positioning of drain droplet with increased sodium oleate content (>10 wt%). Optical microscopy recording at 1.25x magnification of three separate experiments where an oleic acid drain droplet (indicated with the yellow dots) with a larger sodium oleate content was deposited in between two free-floating source droplets (pure $C_{12}E_4OH$, 1.0 μL, indicated with the red dots) at the air-water interface of the sodium alginate solution. The increased sodium oleate content (**a,** 20 wt%; **b,** 15 wt%; **c,** 15 wt%) was observed to decrease the filament absorption capacity compared to a oleic acid drain droplet with 10 wt% sodium oleate (Fig. 6). The scale bar represents 2 mm.

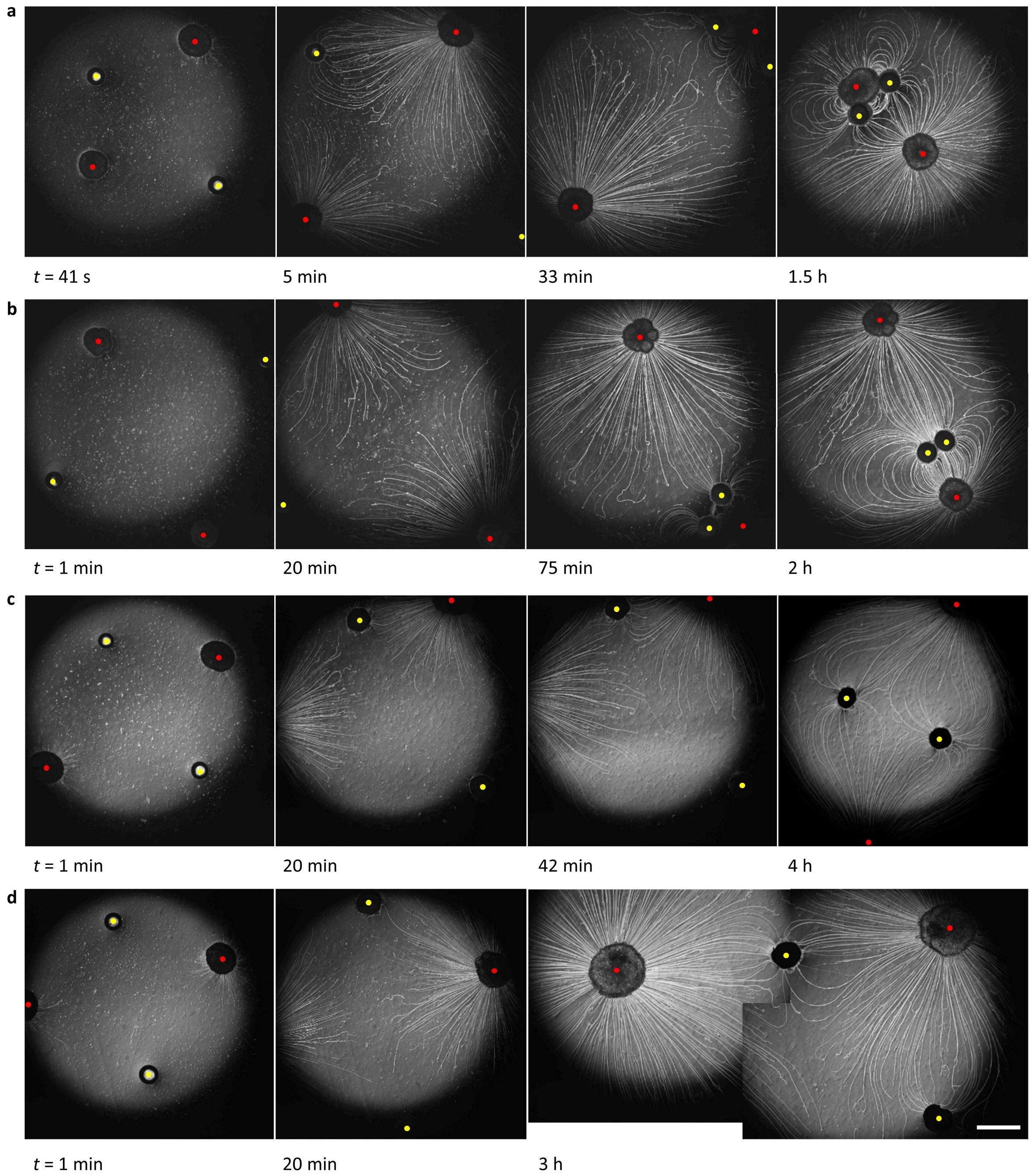

Supplementary Figure 15. Self-organization of a 4-droplet system. Optical microscopy recording at 1.25x magnification of four separate experiments where two drain droplets (sodium oleate in oleic acid, 1.0 µL, indicated with the yellow dots) position themselves with respect to two free-floating amphiphile source droplets (pure $C_{12}E_4OH$, 1.0 µL, indicated with the red dots) at the air-water interface of an aqueous sodium alginate solution. **a,** With 10 wt% sodium oleate, the drains ended up very close to the source. A larger sodium oleate content decreases the filament absorption capacity (Supplementary Fig. 14), and with 11 wt% sodium oleate (**b**) and 15 wt% sodium oleate (**c, d**) in the oleic acid drain, the drains were positioned again further away from the source droplets. The scale bar represents 2 mm.

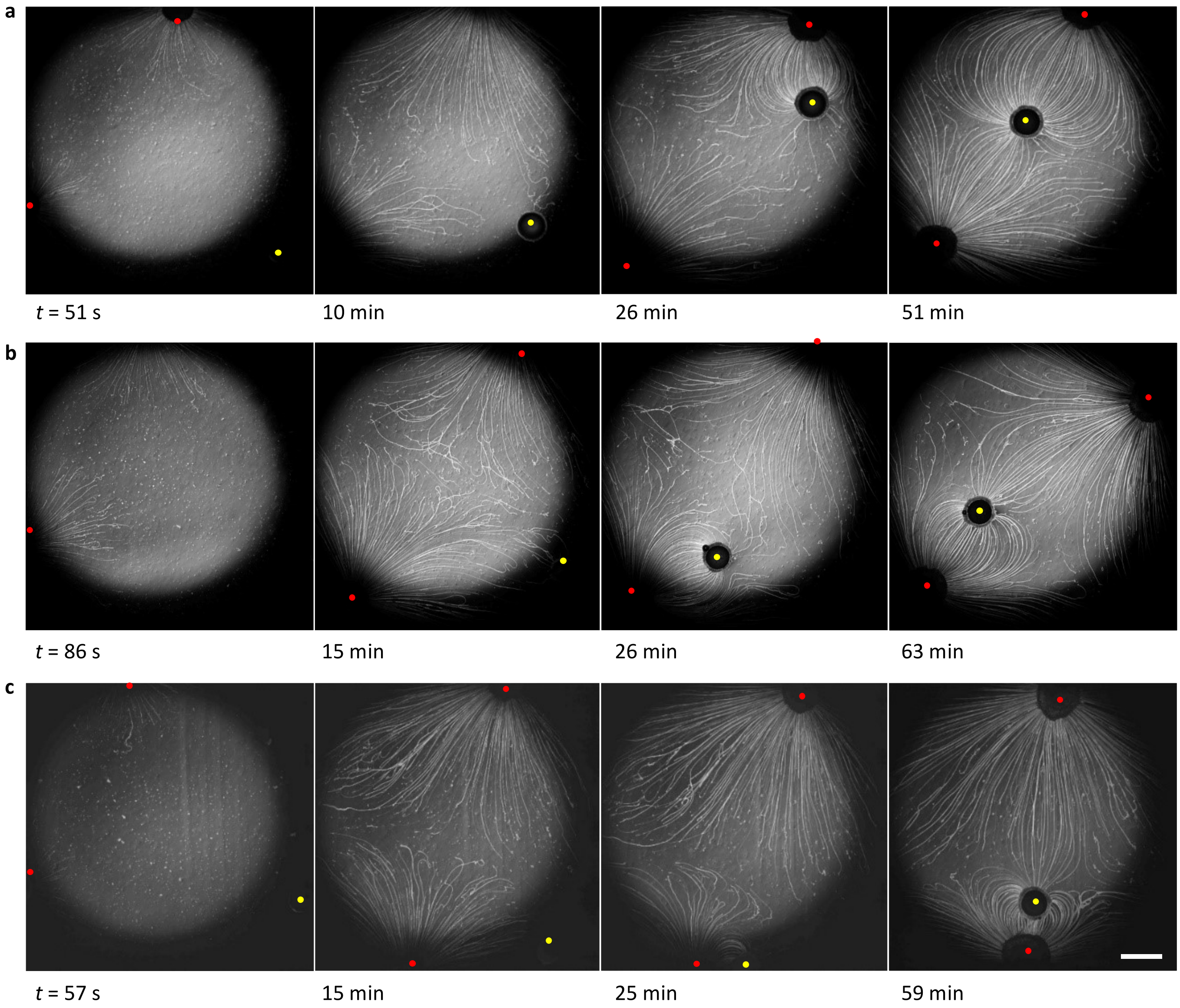

Supplementary Figure 16. Self-positioning of 3-droplet system, from off-centered to linear organization (1). a-c, Optical microscopy recording at 1.25x magnification of 3 separate experiments, conducted under similar conditions, where a drain droplet (10 wt% sodium oleate in oleic acid, 1.0 µL, indicated with the yellow dot) centers itself in between two free-floating amphiphile source droplets (pure $C_{12}E_4OH$, 1.0 µL, indicated with the red dots) at the air-water interface of the sodium alginate solution. After initial deposition of the drain droplet, off-centered to the two source droplets, absorption of the filaments drew the drain to a position in between the two source droplets – in analogy to the results shown in Fig. 6b and Supplementary Fig. 17, which have been acquired under similar conditions. The scale bar represents 2 mm.

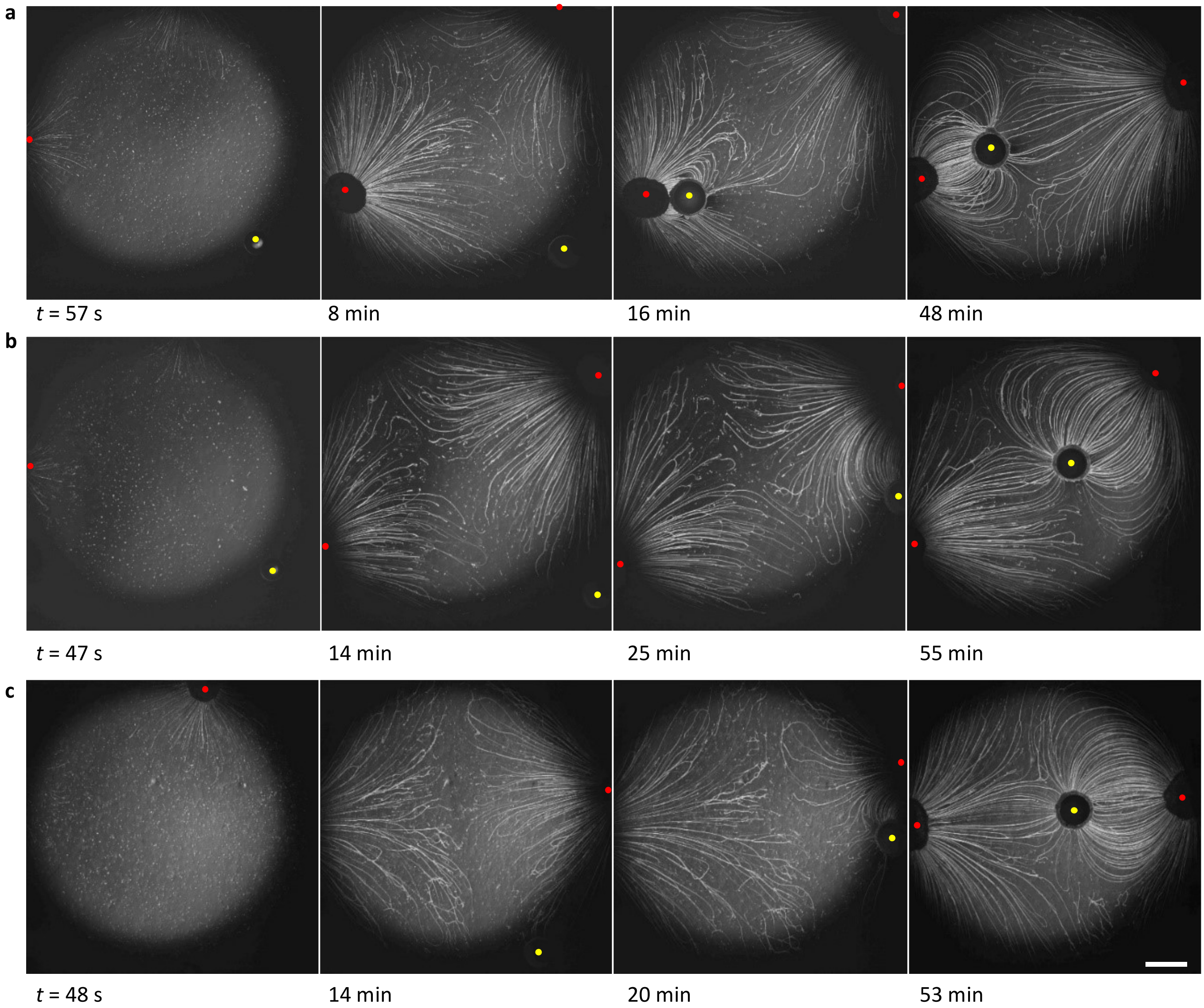

Supplementary Figure 17. Self-positioning of 3-droplet system, from off-centered to linear organization (2). a-c, Optical microscopy recording at 1.25x magnification of 3 separate experiments, conducted under similar conditions, where a drain droplet (10 wt% sodium oleate in oleic acid, 1.0 μL, indicated with the yellow dot) centers itself in between two free-floating amphiphile source droplets (pure $C_{12}E_4OH$, 1.0 μL, indicated with the red dots) at the air-water interface of the sodium alginate solution. After initial deposition of the drain droplet, off-centered to the two source droplets, absorption of the filaments drew the drain to a position in between the two source droplets – in analogy to the results shown in Fig. 6b and Supplementary Fig. 16, which have been acquired under similar conditions. The scale bar represents 2 mm.

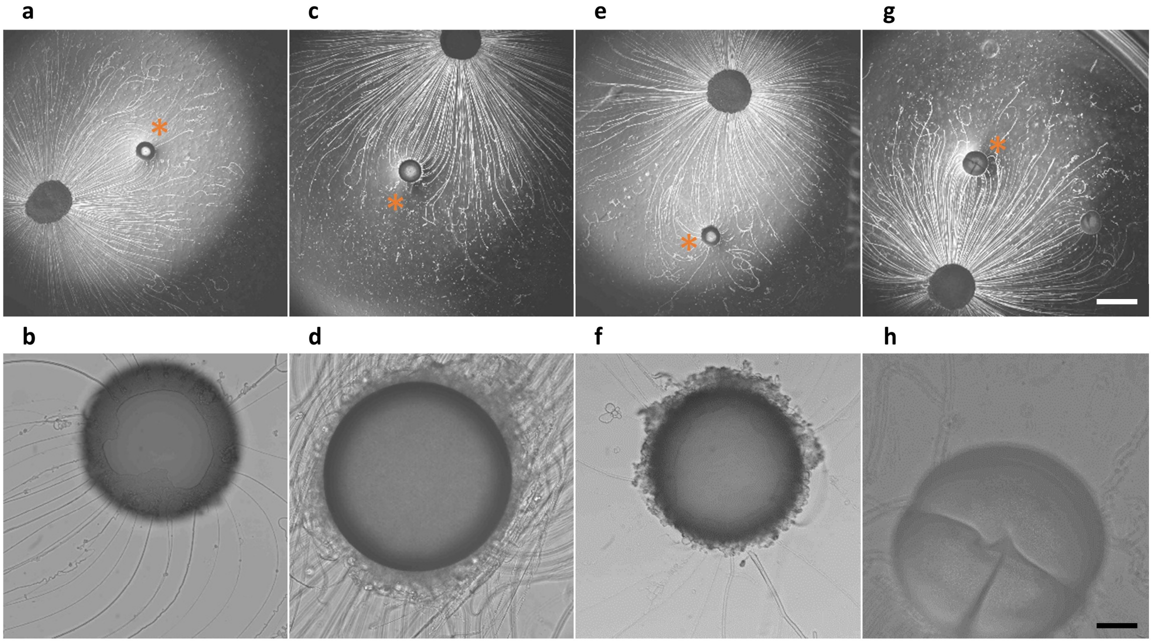

Supplementary Figure 18. Analyzing the effect of sodium salts on drain droplet stability. Optical microscopy recording of self-organization of pure $C_{12}E_4OH$ source droplets (1.0 µL) and 10 wt% sodium oleate in oleic acid drain droplets (1.0 µL, indicated with asterisk). The presence of a sodium salt (**a-b**, 17.3 sodium ascorbate, **c-d** 17.3 mM sodium chloride, **e-f** 17.3 mM sodium benzoate) in the aqueous sodium alginate solution stabilizes the drain droplets in comparison to the absence of an added sodium salt (**g-h**). The images in (a, c, e, g) were acquired at 1.25x magnification (scale bar represents 2 mm); the images in (b, d, f, h) feature the respective drain droplet at 10x magnification (scale bar represents 250 µm).
