# Supplementary material for: Autonomous mesoscale positioning emerging from myelin filament self-organization and Marangoni flows
Source: Nat Commun. 2020 Sep 23;11:4800. doi: 10.1038/s41467-020-18555-w (PMC7511956; doi:10.1038/s41467-020-18555-w)
Supplement: Supplementary file 3 — Descriptions of Additional Supplementary Files [file 41467_2020_18555_MOESM3_ESM.pdf]

Descriptions of Additional Supplementary Files

Supplementary Movie 1

Description: Filament growth from source droplet deposited at air-water interface. Optical microscopy movies corresponding to Figure 1b, c. Left: 0.5 µL of the amphiphile solution was deposited at the air-water interface of an aqueous sodium alginate solution. Right: 1.0 µL of the amphiphile solution was deposited at a metal pin that was inserted in the sodium alginate solution, and the filament growth was followed at a larger magnification (10x).

Supplementary Movie 2

Description: Filament absorption at the drain droplet. Optical microscopy movies corresponding to Figure 1f. 0.5 µL of the amphiphile solution and 0.5 µL of a drain solution (10 wt% sodium oleate in oleic acid) were deposited at the air-water interface of the sodium alginate solution. The left video was acquired at a 4x magnification; the right video at a 10x magnification.

Supplementary Movie 3

Description: Source droplet – drain droplet interaction in 3 different regimes. Optical microscopy movies corresponding to Figures 2e-g. Left: 0.5 µL of the amphiphile solution and 0.5 µL of oleic acid were deposited at the air-water interface of the sodium alginate solution resulting into a merge of the source and drain droplets – following regime 1. Middle: 0.5 µL of the amphiphile solution and 0.5 µL of the drain solution (10 wt% sodium oleate in oleic acid) were deposited, resulting into a repulsion of the source and drain droplets – following regime 2. Right: 0.5 µL of the amphiphile solution was deposited, and subsequently the drain droplet (0.5 µL of 10 wt% sodium oleate in oleic acid) was deposited such that it absorbed the filaments and a stable self-organization was established – following regime 3.

Supplementary Movie 4

Description: Filament absorption generates attractive forces at the drain droplet. Optical microscopy movie corresponding to Figure 3a, showing the self-organization that is established by a pure C12E4OH source droplet (1.0 µL) and two 10 wt% sodium oleate in oleic acid droplets (1.0 µL) that were subsequently deposited at the air-water interface of the sodium alginate solution. The positioning of the drains with respect to the source droplet is maintained by a balance between the Marangoni flow that repels the drain droplet, and filament absorption at the drain that generates attractive forces.

Supplementary Movie 5

Description: Filament absorption generates attractive forces at the drain droplet (2). Optical microscopy movie corresponding to Figure 3d-e: A C12E4OH source droplet (1.0 µL) and an oleic acid drain droplet (1.0 µL) were deposited subsequently at an aqueous sodium alginate solution that included C12E4OH (4.3 mM) to suppress the Marangoni flow from the source droplet. Upon absorption of the filaments, the drain moved abruptly into the direction where the particular

absorption event took place, indicating that absorption of filaments generates attractive forces effective on the drain droplet.

Supplementary Movie 6

Description: Droplet positioning in hexagon with 6 drain droplets. Optical microscopy movie corresponding to Figure 4a: the positioning of a C12E4OH source droplet (1.0 µL) within a hexagon with 6 drain droplets (1.0 µL of 10 wt% sodium oleate in oleic acid). The filaments tethered to the drain droplets, positioned at the tips of the hexagon that was immersed in the sodium alginate solution, and the source droplet maintained its position within the hexagon over the course of more than 1 hour.

Supplementary Movie 7

Description: Autonomous centring in a 3-droplet system. Optical microscopy movie corresponding to Figure 6a; two 1.0 µL pure C12E4OH source droplets were deposited simultaneously at the air-water interface of the sodium alginate solution. After a 25 second delay, a drain droplet (1.0 µL, 10 wt% sodium oleate in oleic acid) was deposited in between these two source droplets. After its initial attraction towards one of the source droplets, the drain droplet started to position itself in the middle of the two source droplets once filaments emerging from the upper source droplet were absorbed at the drain as well.

Supplementary Movie 8

Description: 3 droplets self-organizing into a line. Optical microscopy movie corresponding to Figure 6b; two 1.0 µL pure C12E4OH source droplets were deposited simultaneously at the air-water interface of the sodium alginate solution. After a 25 second delay, a drain droplet (1.0 µL, 10 wt% sodium oleate in oleic acid) was deposited off-centered to these two source droplets. After its initial repulsion from the source droplets due to the Marangoni flow, the drain droplet was attracted towards one of the source droplets as filaments were absorbed by the drain. The drain droplet then positioned itself in between the two source droplets once filaments emerging from the other source droplet were absorbed at the drain droplet as well.
